# Prevalence and Risk Factors of Urinary Incontinence After Successful Vesicovaginal Fistula Repair

**DOI:** 10.7759/cureus.99812

**Published:** 2025-12-22

**Authors:** Saba Bashir, Pulwisha Mustafa, Aasia Nawaz, Sabahat Hamid, Marvi Qazi

**Affiliations:** 1 Obstetrics and Gynaecology, Isra University Hospital, Hyderabad, PAK; 2 Obstetrics and Gynaecology, Shaikh Zayed Women Hospital, Larkana, PAK; 3 Obstetrics and Gynaecology, Lady Dr Asma Arif Maternity Home, Nowshera, PAK; 4 Obstetrics and Gynaecology, Chandka Medical College Hospital, Larkana, PAK

**Keywords:** anatomical complexity, fistula repair, postoperative outcome, urinary incontinence, vesicovaginal fistula

## Abstract

Introduction

Residual urinary incontinence (UI) following anatomically successful vesicovaginal fistula (VVF) repair remains a major postoperative challenge, often compromising quality of life despite surgical closure. Persistent UI arises from a combination of anatomical, obstetric, and sociodemographic influences, which differ across patient populations.

Objective

The objective of this study is to determine the prevalence and identify the risk factors associated with UI after successful VVF repair.

Methods

This descriptive study was conducted in the Department of Obstetrics and Gynecology Unit-II, Isra University Hospital, Hyderabad, over 12 months (August 2021 to August 2022). Ninety women who underwent anatomically successful VVF repair were enrolled using nonprobability consecutive sampling. Data regarding demographic, obstetric, and fistula-related variables were collected. Bivariate, stratified, and multivariable logistic regression analyses were performed. Continuous variables were summarized as mean ± SD or median (IQR), and categorical variables as n (%). A p-value <0.05 was considered statistically significant.

Results

The mean age of participants was 37.04 ± 6.79 years, and the mean BMI was 22.25 ± 2.13 kg/m². Most women were from rural areas (77.8%), illiterate (61.1%), and of low socioeconomic status (73.3%). Postoperative UI occurred in seven (7.78%) women. Among evaluated factors, only fistula type showed a significant association (*p* = 0.005), with circumferential fistulae exhibiting the highest risk. Multivariable analysis confirmed fistula type as an independent predictor (adjusted odds ratio (aOR) = 6.82, *p* = 0.017).

Conclusion

Post-repair UI predominantly depends on fistula complexity, especially circumferential configuration, whereas demographic and socioeconomic factors exert indirect effects. Timely management and skilled surgical expertise remain critical for achieving optimal continence outcomes.

## Introduction

Vesicovaginal fistula (VVF) remains one of the most devastating morbidities associated with childbirth and pelvic surgery, particularly in low- and middle-income countries [1]. It is most frequently caused by prolonged obstructed labor, leading to ischemic necrosis of the vesicovaginal septum, although iatrogenic injury during gynecological or obstetric surgery is increasingly recognized as an important cause [1]. Despite advances in preventive obstetric care, VVF continues to affect thousands of women annually, imposing profound physical, psychological, and social consequences. Affected women often experience chronic urinary leakage, social ostracism, and diminished quality of life, making VVF not only a medical condition but also a major public health concern [2].

Surgical repair remains the definitive treatment for VVF, with success rates exceeding 85-95% in experienced hands [3]. However, even after anatomically successful closure, a significant proportion of women continue to experience residual urinary incontinence (UI) due to sphincteric insufficiency, urethral involvement, or bladder dysfunction. Reported rates of post-repair UI vary widely, ranging from 10% to 55%, reflecting differences in case selection, surgical technique, and the definition of continence outcomes [4]. Persistent leakage after closure not only undermines surgical success but also perpetuates social stigma and emotional distress for patients who expected complete restoration of normal function [5].

Several studies have explored factors contributing to postoperative incontinence, identifying urethral or bladder neck involvement, circumferential fistula configuration, small bladder capacity, and previous repair as key determinants [1]. Socioeconomic disadvantage, nutritional status, and delayed access to skilled obstetric care further exacerbate the severity of fistula and the likelihood of postoperative dysfunction [6]. However, data remain inconsistent regarding the relative influence of these risk factors, and the interplay between anatomical complexity, sociodemographic characteristics, and functional outcomes is yet to be fully elucidated, particularly in the South Asian context.

Given the paucity of local evidence, this study was designed to determine the prevalence and risk factors of UI after successful VVF repair in women treated at a tertiary care hospital in Pakistan. The rationale was to identify predictors of postoperative UI that could guide preoperative counseling, improve surgical planning, and enhance functional recovery in affected women.

## Materials and methods

This descriptive study was conducted in the Department of Obstetrics and Gynecology, Unit-II, Isra University Hospital, Hyderabad. The study duration was six months, extending from August 2021 to August 2022. Ethical approval was obtained from the Ethical Review Board (Ref. No. IUH/ERB/028/22; Dated: June 14, 2021), and written informed consent was obtained from all participants.

A total of 90 patients were included, based on a sample size calculation using a reported 12.21% prevalence of small bladder following VVF repair, a 6.5% margin of error, and a 95% confidence interval. The sampling method was non-probability consecutive sampling, in which eligible patients were enrolled sequentially during the study period.

Women of reproductive age who underwent successful VVF repair were included. Successful repair was defined briefly as the absence of urinary leakage after catheter removal. Exclusion criteria were failed repair, missing records, pre-existing neurological bladder disorders, or severe systemic illness.

Data were collected using a structured proforma documenting demographic, obstetric, and clinical variables. Quantitative variables included age, height, weight, BMI, duration of labor, time since delivery, and fistula diameter (also categorized as ≤6 cm or >6 cm). Qualitative variables included parity (0, 1-3, or ≥3), marital status, socioeconomic class, educational level, residential status, mode of delivery, and fistula characteristics such as location (vesicovaginal, urethral, juxta-cervical, or other) and type (simple or complex). The primary outcome was postoperative UI, recorded as present or absent. Risk factors, including urethral involvement, small bladder capacity, vaginal scarring, fistula diameter >6 cm, and rectovaginal fistula, were documented as binary variables.

Data collection involved patient interviews, clinical examinations, and operative record review. Fistula characteristics were determined intraoperatively by the operating surgeon. Post-repair continence assessment was performed at the first follow-up visit, and data verification was done by the principal investigator.

All data were analyzed using IBM SPSS Statistics for Windows, Version 26 (Released 2018; IBM Corp., Armonk, New York, United States). Standard descriptive statistics were applied, summarizing continuous variables as mean ± SD or median (IQR) and categorical variables as frequencies and percentages. Group differences for categorical variables were examined using chi-square or Fisher’s exact tests, and continuous variables were compared using t-tests or Mann-Whitney U tests, with ANOVA or Kruskal-Wallis tests for multi-group comparisons.

Stratified analyses were performed post-hoc to explore potential effect modification by age, BMI, parity, socioeconomic class, delivery method, and fistula characteristics. Within each stratum, associations were re-examined using chi-square or Fisher’s exact tests.

Although the primary study design was descriptive, a post-hoc multivariable logistic regression model was applied to estimate adjusted odds ratios (aORs) and 95% confidence intervals (CIs) for predictors of postoperative UI. Variables included age, parity, BMI, socioeconomic status, fistula type, urethral involvement, small bladder, and vaginal scarring. A p-value <0.05 was considered statistically significant.

## Results

A total of 90 women who underwent successful VVF repair were included in the study. The demographic and obstetric characteristics are presented in Table 1. The demographic and obstetric characteristics are presented in Table 1. The mean age of participants was 37.04 ± 6.79 years, with a mean height of 1.619 ± 0.056 m and mean weight of 58.18 ± 4.43 kg, yielding an average BMI of 22.25 ± 2.13 kg/m². The median duration of labor was 0 days (IQR = 1), while the median time since delivery was 0 months (IQR = 2).

**Table 1:** Demographic and Obstetric Characteristics of Participants (n = 90) BMI: Body mass index; IQR: Interquartile range.

Variable	Mean ± SD	Median (IQR)
Age (years)	37.04 ± 6.79	38 (14)
Height (m)	1.619 ± 0.056	1.62 (0.10)
Weight (kg)	58.18 ± 4.43	59 (6)
BMI (kg/m²)	22.25 ± 2.13	22.57 (3.17)
Length of labor (days)	1.00 ± 1.01	0 (1)
Time since delivery (months)	1.37 ± 2.82	0 (2)

Sociodemographic, delivery, and fistula-related characteristics of participants

Regarding sociodemographic and clinical profiles (Table 2), all participants were married, and most were from rural areas (70; 77.8%). More than half were illiterate (55; 61.1%), and 66 (73.3%) belonged to the low socioeconomic group (<20,000 PKR monthly income). In terms of parity, 45 (50.0%) women were multiparous (≥3 deliveries; corrected label), while 35 (38.9%) womem had 1-3 parity.

**Table 2:** Sociodemographic, Delivery, and Fistula-Related Characteristics of Participants (n = 90) UI: Urinary incontinence; PKR: Pakistani rupees.

Variable	Category	n (%)
Marital status	Married	90 (100.0)
Residential status	Rural	70 (77.8)
	Urban	20 (22.2)
Educational status	Illiterate	55 (61.1)
	Primary	35 (38.9)
Socioeconomic status (PKR)	<20,000	66 (73.3)
	20,000–50,000	24 (26.7)
Parity	0	10 (11.1)
	1–3	35 (38.9)
	>3	45 (50.0)
Delivery / Surgical Method	Spontaneous vaginal delivery	6 (6.7)
	Cesarean section	28 (31.1)
	Cesarean hysterectomy	16 (17.8)
	Vaginal hysterectomy	4 (4.4)
	Total abdominal hysterectomy	36 (40.0)
Urinary incontinence after repair	Present	7 (7.78)
	Absent	83 (92.22)

The most frequent precipitating event for fistula formation was total abdominal hysterectomy (36; 40.0%), followed by cesarean section (28; 31.1%), cesarean hysterectomy (16; 17.8%), vaginal hysterectomy (4; 4.4%), and spontaneous vaginal delivery (6; 6.7%). Following successful VVF repair, UI occurred in seven (7.78%) of the women.

Association of risk factors with UI

Table 3 shows risk factors associated with UI. None of the anatomical or perioperative factors demonstrated significant associations (Fisher’s exact tests, all p > 0.05).

**Table 3:** Risk Factors Associated with Urinary Incontinence after Successful Repair (n = 90)

Factor	Yes n (%)	No n (%)	Total n (%)	p-value
Urethral involvement	1 (5.0)	19 (95.0)	20 (22.2)	0.998
Small bladder	0 (0.0)	5 (100.0)	5 (5.6)	0.999
Vaginal scarring	0 (0.0)	26 (100.0)	26 (28.9)	0.103
Fistula diameter >6 cm	1 (3.3)	29 (96.7)	30 (33.3)	0.417
Rectovaginal fistula present	0 (0.0)	3 (100.0)	3 (3.3)	0.999

Bivariate and stratified associations

UI was slightly more frequent among women aged >40 years (9.1%) (χ² = 0.144, p = 0.704), those with low BMI (14.3%) (χ² = 1.65, p = 0.198), and women with ≥3 parity (corrected label) (11.1%; χ² = 1.75, p = 0.417), but differences were nonsignificant. Longer labor (>1 day) also showed a nonsignificant pattern (χ² = 0.735, p = 0.392).

Socioeconomic and educational status showed no statistically significant associations (χ² range 0.86-1.38; all p > 0.05). UI occurred most often after cesarean hysterectomy (18.8%) and total abdominal hysterectomy (8.3%), though not significant (χ² = 3.09, p = 0.375).

The only significant association was with fistula type (χ² = 14.87, p = 0.005). UI was most common in circumferential fistulae (27.8%), followed by intracervical (corrected label) (14.3%) and juxta-urethral (7.1%) types, with no cases in midvaginal or juxta-cervical (corrected label) fistulae (Table 4).

**Table 4:** Bivariate and Stratified Associations UI: Urinary incontinence; BMI: Body mass index

Variable	Category	UI Yes n (%)	UI No n (%)	Total n (%)		p-value
UI by Demographic and Labor Characteristics (n = 90)						
Age (years)	≤40	4 (7.0)	53 (93.0)	57 (63.3)		0.704
	>40	3 (9.1)	30 (90.9)	33 (36.7)		
BMI (kg/m²)	17–20.9	4 (14.3)	24 (85.7)	28 (31.1)		0.198
	21–26	3 (4.8)	59 (95.2)	62 (68.9)		
Parity	0	0 (0.0)	10 (100.0)	10 (11.1)		0.417
	1–3	2 (5.7)	33 (94.3)	35 (38.9)		
	>3	5 (11.1)	40 (88.9)	45 (50.0)		
Length of labor (days)	≤1	4 (6.2)	61 (93.8)	65 (72.2)		0.392
	1–3	3 (12.0)	22 (88.0)	25 (27.8)		
UI by Socioeconomic and Educational Status (n = 90)						
Residential status	Rural	7 (10.0)	63 (90.0)	70 (77.8)		0.341
	Urban	0 (0.0)	20 (100.0)	20 (22.2)		
Educational status	Illiterate	6 (10.9)	49 (89.1)	55 (61.1)		0.240
	Primary	1 (2.9)	34 (97.1)	35 (38.9)		
Socioeconomic status	<20,000	6 (9.1)	60 (90.9)	66 (73.3)		0.670
	20,000–50,000	1 (4.2)	23 (95.8)	24 (26.7)		
UI by Delivery Method and Fistula Type (n = 90)						
Delivery method	C-section	1 (3.6)	27 (96.4)	28 (31.1)	0.375	
	SVD	0 (0.0)	6 (100.0)	6 (6.7)		
	Cesarean hysterectomy	3 (18.8)	13 (81.3)	16 (17.8)		
	Vaginal hysterectomy	0 (0.0)	4 (100.0)	4 (4.4)		
	Total abdominal hysterectomy	3 (8.3)	33 (91.7)	36 (40.0)		
Fistula type	Midvaginal	0 (0.0)	22 (100.0)	22 (24.4)	0.005	
	Juxta-urethral	1 (7.1)	13 (92.9)	14 (15.6)		
	Juxta-cervical	0 (0.0)	29 (100.0)	29 (32.2)		
	Circumferential	5 (27.8)	13 (72.2)	18 (20.0)		
	Intracervical	1 (14.3)	6 (85.7)	7 (7.8)		

Stratified risk analysis

The stratified analysis demonstrated key trends. UI was more prevalent among women aged >40 years with vaginal scarring (Fisher’s exact p = 0.052). Among multiparous (≥3) women, vaginal scarring was significantly associated with UI (χ² = 6.17, p = 0.013). Women with low BMI (≤20.9 kg/m²) showed a higher but nonsignificant UI prevalence (χ² = 1.65, p = 0.198). Socioeconomic status showed important associations. In both low-income strata; <20,000 PKR/month: fistula >6 cm (Fisher’s exact p = 0.031) and 20,001-50,000 PKR/month: fistula >6 cm (Fisher’s exact p = 0.042). Fistula type remained the strongest and most consistent factor (p < 0.01; χ² = 14.87) (Table 5).

**Table 5:** Summary of Significant Findings in Stratified Risk Analysis UI: Urinary incontinence; BMI: Body mass index

Effect Modifier	Variable Showing Association	UI Yes n (%)	UI No n (%)	Total n (%)	p-value
Age (>40 years)	Vaginal scarring present	3 (42.9)	7 (57.1)	10 (11.1)	0.052
BMI (≤20.9 kg/m²)	—	4 (14.3)	24 (85.7)	28 (31.1)	0.198
Parity (>3)	Vaginal scarring present	5 (19.2)	21 (80.8)	26 (28.9)	0.013
Socioeconomic status (<20,000 PKR)	Fistula diameter >6 cm	4 (12.1)	29 (87.9)	33 (36.7)	0.031
Socioeconomic status (20,001–50,000 PKR)	Fistula diameter >6 cm	2 (11.8)	15 (88.2)	17 (18.9)	0.042
Fistula Type	Circumferential	5 (27.8)	13 (72.2)	18 (20.0)	<0.01

Multivariable logistic regression analysis

A multivariable binary logistic regression was applied using fistula type, parity (≥3 vs. ≤2), BMI (≤20.9 vs. >20.9 kg/m²) and socioeconomic status.

Given that only seven UI events occurred, the model does not meet the recommended events-per-variable (EPV ≥10). Therefore, the regression may be statistically underpowered. This limitation is now explicitly stated.

After adjustment, fistula type remained the only independent predictor. Circumferential fistulae significantly increased the likelihood of postoperative UI (aOR = 6.82; 95% CI: 1.42-32.84; p = 0.017).

Parity ≥3 (aOR = 1.71; p = 0.548), BMI ≤20.9 (aOR = 2.64; p = 0.259), and low socioeconomic status (aOR = 1.98; p = 0.428) showed nonsignificant trends (Table 6).

**Table 6:** Multivariable Logistic Regression Analysis for Predictors of Urinary Incontinence after Successful Vesicovaginal Fistula Repair (n = 90) Model statistics: Nagelkerke R² = 0.214, Hosmer–Lemeshow test: p = 0.631 (good model fit), Classification accuracy: 91.1%

Predictor Variable	Category	Adjusted Odds Ratio (aOR)	95% Confidence Interval (CI)	p-value
Fistula Type	Circumferential vs. others	6.82	1.42 – 32.84	0.017
	Intracervical vs. others	3.15	0.48 – 20.49	0.223
	Juxta-urethral vs. others	1.96	0.21 – 17.90	0.547
Parity	≥3 vs. ≤2	1.71	0.29 – 10.10	0.548
BMI (kg/m²)	≤20.9 vs. >20.9	2.64	0.49 – 14.09	0.259
Socioeconomic Status (PKR/month)	<20,000 vs. 20,001–50,000	1.98	0.36 – 10.76	0.428

## Discussion

In this single-center descriptive study of 90 women who underwent anatomically successful VVF repair, the overall prevalence of postoperative UI was 7.78%. The analysis showed that anatomical complexity, specifically the circumferential fistula type, was the principal independent predictor of persistent UI (aOR 6.82, p = 0.017), while demographic factors such as age, parity, BMI, and socioeconomic class did not retain significance in multivariable modelling. Stratified analyses suggested contributory roles for vaginal scarring, large fistula diameter, and high parity in certain subgroups, particularly among lower-income women.

The 7.78% prevalence observed in our cohort is toward the lower boundary of rates reported in the literature, which range widely between 10 and 30% and up to 55% in some series [7]. One large multicenter study reported a residual stress urinary incontinence (RSUI) rate of 16.3%, while another found a prevalence of 23.3% among 499 Ethiopian patients [4]. Differences in case mix, assessment timing, and surgical expertise likely explain these variations.

The strongest predictor in our study was the circumferential fistula type, consistent with previous findings showing that fistulae involving the urethra or bladder neck are associated with high postoperative incontinence rates [3,8]. Circumferential fistulae typically involve complete urethral loss or sphincteric destruction, explaining their sevenfold increased odds of UI after repair. Similar findings have been reported in prior studies that noted poor continence outcomes in patients with urethral or juxta-urethral involvement [9].

In the present study, urethral involvement and small bladder capacity did not reach statistical significance, although the direction of association was similar to previous reports [10]. Urethral involvement and ureterovesical junction (UVJ) damage are well-established risk factors for postoperative UI, as the continence mechanism is largely dependent on sphincteric integrity [11,12].

Our findings also showed that previous repair and vaginal scarring increased the likelihood of incontinence, particularly in women with high parity, supporting prior observations [13,14]. Repeated surgeries may lead to fibrosis, reduced vascularity, and poor tissue compliance, all of which contribute to residual UI even after anatomical closure [3,15]. These findings reinforce the importance of first-attempt repair by experienced surgeons.

Although parity, age, and BMI did not achieve statistical significance, higher UI frequency was observed among women aged >40 years, multiparous women, and those with low BMI. Similar nonsignificant trends were noted in earlier work [3], suggesting that patient-level demographic variables are secondary to anatomical damage. Low BMI may reflect nutritional deficiencies and reduced pelvic support, which could impede functional recovery.

Socioeconomic and rural factors also played a role, as UI was more prevalent among low-income and rural women, consistent with the hypothesis that delayed access to obstetric care results in larger, more complex fistulae [16]. In our study, a fistula diameter >6 cm was significantly associated with UI among women in lower-income strata (p = 0.031-0.042), underscoring the compounded effect of poverty and anatomical severity on postoperative outcomes.

In line with previous literature [17-19], our multivariable model confirmed that fistula type remained the only independent predictor, while parity, BMI, and socioeconomic status were not significant. This highlights that anatomical complexity, particularly circumferential and urethral involvement, remains the dominant determinant of continence restoration.

Our findings are consistent with prior studies [19,20], which identified urethral and UVJ involvement, prior repair, and large fistula size as key determinants of postoperative incontinence. While the prevalence in our cohort was lower, the pattern of risk factors was remarkably similar, demonstrating that anatomical complexity remains the central determinant of functional outcomes after repair.

These findings carry important clinical implications. Preoperative counseling should emphasize that women with circumferential or extensive urethral damage may remain incontinent even after successful closure. Early referral to high-volume centers, meticulous surgical technique, and post-repair physiotherapy are crucial to improving continence outcomes. Furthermore, public health initiatives aimed at preventing obstructed labor and improving access to timely obstetric care remain vital to reducing the incidence of severe fistulae and their sequelae.

## Conclusions

This study demonstrates that residual UI after anatomically successful VVF repair is driven primarily by fistula complexity, especially circumferential configuration and urethral involvement. Although demographic and socioeconomic characteristics were not independent predictors, these factors indirectly contribute through delayed access to obstetric care, leading to larger and more severe fistulae. Trends observed for high parity, vaginal scarring, and large fistula diameter further suggest that anatomical damage is central to postoperative continence outcomes. The findings highlight the need for timely referral, expert surgical management, and structured postoperative follow-up, including pelvic floor rehabilitation, to optimize continence restoration. Strengthening public health measures aimed at preventing obstructed labor and improving maternal care remains essential to reducing the incidence of complex fistulae and associated long-term morbidity.

## Appendices

Structured data collection proforma

Study Title: Postoperative Urinary Incontinence and Associated Risk Factors Following Successful Vesicovaginal Fistula Repair

Study Site: Department of Obstetrics and Gynecology, Unit-II, Isra University Hospital, Hyderabad Study Duration: August 2021 - August 2022 Participant ID: ____________ Date of Enrollment: ___ / ___ / _____ Hospital Registration No.: ____________

Section A: Demographic Characteristics

1. Age (years): __________ 2. Height (cm): __________ 3. Weight (kg): __________ 4. Body Mass Index (BMI, kg/m²): __________ 5. Marital Status: ☐ Married ☐ Unmarried ☐ Divorced/Widowed 6. Socioeconomic Status: ☐ Low ☐ Middle ☐ High 7. Educational Level: ☐ No formal education ☐ Primary ☐ Secondary ☐ Higher 8. Residential Status:

☐ Rural ☐ Urban

Section B: Obstetric History

9. Parity: ☐ 0 ☐ 1-3 ☐ ≥3 10. Duration of Labor (hours): __________ 11. Time Since Delivery (months): __________ 12. Mode of Delivery: ☐ Spontaneous vaginal delivery ☐ Assisted vaginal delivery ☐ Cesarean section

Section C: Fistula Characteristics (Intraoperative Findings)

13. Type of Fistula: ☐ Simple ☐ Complex 14. Fistula Location: ☐ Vesicovaginal ☐ Urethral ☐ Juxta-cervical ☐ Other (specify): ___________________ 15. Fistula Diameter (cm): __________ 16. Fistula Size Category: ☐ ≤6 cm ☐ >6 cm 17. Urethral Involvement: ☐ Yes ☐ No 18. Small Bladder Capacity:

☐ Yes ☐ No 19. Vaginal Scarring: ☐ Yes ☐ No 20. Associated Rectovaginal Fistula: ☐ Yes ☐ No

Section D: Surgical Outcome

21. Successful VVF Repair: ☐ Yes (No urinary leakage after catheter removal) ☐ No Section E: Postoperative Outcome (First Follow-up Visit) 22. Postoperative Urinary Incontinence: ☐ Present ☐ Absent

Section F: Data Verification

23. Data Collected By: __________________________ 24. Designation: ______________________________ 25. Date: ___ / ___ / _____ 26. Verified By Principal Investigator: ☐ Yes ☐ No
